# Atrial Fibrillation With Myocardial Infarction Presenting as a Progressive Worsening Fatigue in a Young Male

**DOI:** 10.7759/cureus.26719

**Published:** 2022-07-10

**Authors:** Arjun Mainali, Samaj Adhikari, Amulya Bellamkonda, Tutul Chowdhury, Nicole Gousy, Amita Arora, Alix Dufresne

**Affiliations:** 1 Internal Medicine, Interfaith Medical Center, Brooklyn, USA; 2 Medicine, American University of Antigua, New York, USA; 3 Medicine, Interfaith Medical Center, Brooklyn, USA; 4 Cardiology, Interfaith Medical Center, Brooklyn, USA

**Keywords:** fatigue, atrial fibrillation, myocardial infarction, acute coronary syndrome (acs) and stemi, undiagnosed atrial fibrillation, acute myocardial infarction, chronic fatigue

## Abstract

Atrial fibrillation (AF) is one of the most common arrhythmia exhibiting a dramatic rise in prevalence with associated increased risk of stroke, heart failure, and death. No standard symptoms have been categorized yet to set a gold standard in diagnosing this clinical attribute. A highly variable symptoms array has increased the challenges of management in terms of AF. An obvious relationship has not been established between symptoms and the onset or recurrence of arrhythmia. We present a case of a 43-year-old male patient who complained of chronic fatigue as a primary symptom and was diagnosed with AF with myocardial infarction.

## Introduction

Atrial fibrillation (AF) is the most common cardiac arrhythmia encountered. The health burden of AF is increasing in the USA with an estimation that 12.1 million people will have AF by 2050 [1]. AF significantly increases the risk of stroke; however, AF leading to acute coronary syndrome is rarely discussed. AF leading to myocardial ischemia could be fatal. AF presenting as fatigue may be misleading and can delay the diagnosis and lead to undertreatment. Further, AF is a condition that has been shown to be directly predisposing to acute coronary syndrome [2,3].

We present a case of chronic fatigue in a patient presenting with AF and acute myocardial injury. Our report highlights the bidirectional relationship between AF and myocardial ischemia.

## Case presentation

A 43-year-old male with no significant past medical history presented to the emergency department (ED) after he was referred by his primary medical doctor for tachycardia and elevated blood pressure. He complained of progressive chronic fatigue and occasional palpitations for seven to eight months. He had progressive severe worsening of fatigue for two to three days impairing his daily routine activities, which prompted him to seek medical attention. He denied chest pain, shortness of breath, cough, orthopnea, paroxysmal nocturnal dyspnea, joint or muscle pain, rash, diarrhea, constipation, heat or cold intolerance, and change in appetite and weight. He also denied recent stress, depressed mood, and changes in his sleep pattern. Other reviews of systems were negative otherwise. He denied smoking, use of alcohol, and illicit drugs. On review of his family medical history, he denied any history of premature atherosclerosis or unprovoked arterial embolism.

**Figure 1:**
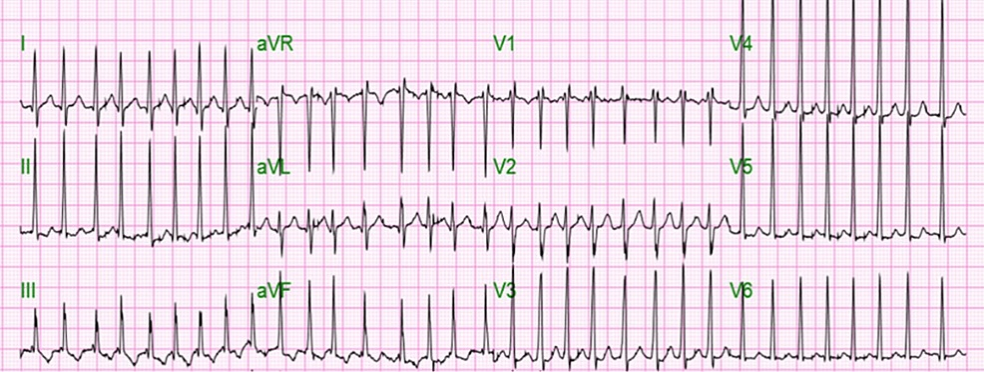
Twelve-lead EKG done during the time of admission showing atrial fibrillation with a heart rate of 200 bpm and no significant ST/T wave changes.

On physical examination, he was not in apparent distress and had a BMI of 24. At triage, his vital signs were blood pressure of 174/124 mmHg and a heart rate of 188 beats/minute. His arterial pulse was irregular. There were no signs of jugular venous distension. A cardiovascular exam revealed tachycardia with irregular rhythm and the absence of any murmurs, rubs, or gallops. A chest exam showed normal breathing with no added breath sounds. All other physical findings were unremarkable. EKG done at the time of admission showed AF with a rapid ventricular response (RVR) with a heart rate of 200 beats/minute and no significant ST/T wave changes, as shown in Figure 1.

The laboratory results showed an elevated high sensitivity troponin of 1902 ng/L, creatine kinase (CK) of 290.5 U/L, urea of 14.6 mg/dl, creatinine of 1.97 mg/dl, and hemoglobin A1c of 6.2. All other laboratory tests, including the comprehensive metabolic panel, complete blood count, thyroid-stimulating hormone, T4, B-type natriuretic peptide, lipid profiles, coagulation profiles, and urine analysis, were within normal limits. Hepatitis B surface antigen (HbsAg), anti-hepatitis C virus (anti-HCV), coronavirus disease 2019 (COVID-19), and urine toxicology were negative. The chest X-ray was also normal. He was also investigated for a cause of secondary hypertension. Serum aldosterone/renin activity, serum cortisol, and metanephrine levels were not significant. His autoimmune workup, including antinuclear antibody (ANA), ribonucleoprotein (RNP) antibodies, Smith antibodies, antiscleroderma-70 antibodies, anti-Sjögren's syndrome-related antigen A, anti-Sjögren's syndrome-related antigen B, and anti-ribosomal P antibodies, was also negative. He received one dose of Cardizem 25 mg and was started on Cardizem drip, aspirin, losartan, clopidogrel, statin, and a therapeutic dose of enoxaparin (1 mg/kg). High sensitivity troponin trended up to 20,074 and then to 24,984 ng/l. An echocardiogram showed left ventricular hypertrophy with normal systolic function with ejection fraction (EF) of 70-75% with no regional wall motion abnormalities.

**Figure 2:**
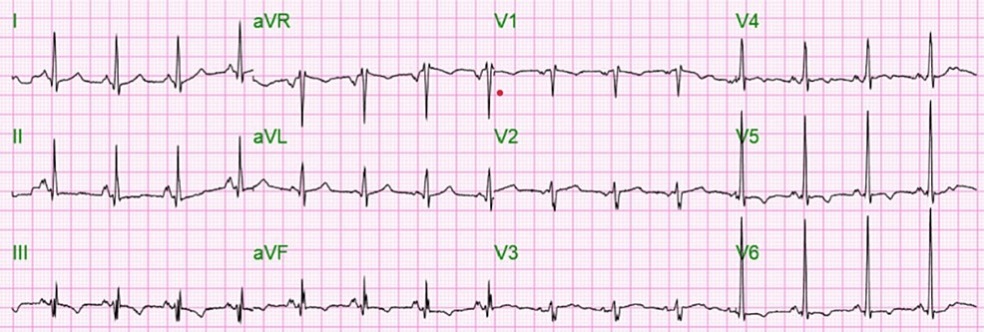
Twelve-lead EKG showing normal sinus rhythm with a heart rate of 90 bpm and nonsignificant ST/T wave changes in leads III, V4, and V6.

His heart rate and blood pressure improved after 12-14 hours, and the Cardizem drip was changed to oral metoprolol. The patient reverted to sinus rhythm within one day, as shown in Figure 2, and his renal functions normalized.

**Figure 3:**
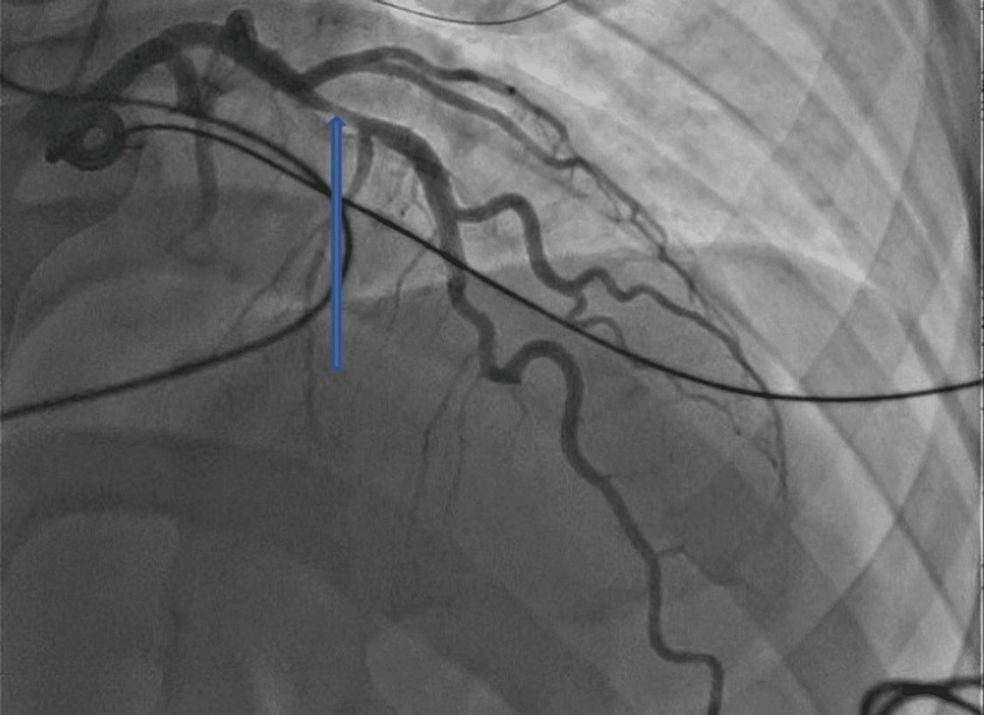
Coronary angiogram showing obstruction in the left anterior coronary artery, as shown by the blue arrow.

He underwent a coronary angiogram on the next day, which showed a left anterior descending artery (LAD) with 70% stenosis, as shown in Figure 3.

The coronary angiogram reports of LAD stenosis could be a subjective finding, and to know the impact of stenotic lesion on the myocardium, he was scheduled for a cardiac stress test, but he refused it even after extensive counseling. His CHA2DS2-VASc (congestive heart failure, hypertension, age ≥ 75 years, diabetes mellitus, stroke or transient ischemic attack, vascular disease, age 65 to 74 years, and sex category) score was 2, and enoxaparin sodium was changed to apixaban after three days. He was discharged with oral Eliquis, aspirin, clopidogrel, atorvastatin, amlodipine, and metoprolol. He was advised to follow up with a cardiologist for a fractional flow reserve (FFR) measurement study and possible percutaneous coronary intervention (PCI) upon discharge. On the follow-up to another hospital, he refused FFR/PCI but had a nuclear pharmacological cardiac stress test, which was negative.

## Discussion

AF is one of the most common cardiac arrhythmias; it currently affects more than five million individuals in the United States and is projected to reach a prevalence of 12.1 million by 2050 [1]. Despite its high prevalence within the community, there is a striking paucity of data regarding the mechanisms by which AF causes these wide variety of symptoms; therefore, diagnosing AF based on symptoms alone can be challenging [4,5]. This is especially worrisome as symptoms are the main predictor of hospitalizations among individuals with AF [6]. In the Framingham Heart Study, the mortality of patients with new-onset AF was reported to be 1.5 (1.2-1.8) in men and 1.9 (1.5-2.2) in women with a 95% confidence interval [7]. One of the largest contributors to mortality in these patients is AF-induced acute myocardial infarction independent of pre-existing comorbidities [3].

Studies have shown that when AF occurs, there is a sustained prothrombotic and inflammatory environment through the activation of platelets, the generation of thrombin, and the induction of endothelial dysfunction, which can exponentially increase the risk for myocardial infarction [2]. This, in conjunction with intermittent episodes of tachycardia, such as seen in this patient, can induce a demand-supply mismatch and further induce myocardial infarction [8]. This creates a bidirectional relationship between myocardial infarction and AF, which can be attributed to the similar risk factors and disease pathogenesis between the two conditions [2].

The risk of AF-induced myocardial infarction is especially elevated in Black and women patients. In the Reasons for Geographic and Racial Differences in Stroke (REGARDS) study, they found that Blacks and women are less likely to be aware of having AF, either due to lack of access to health care or due to symptoms being overlooked or attributed to other chronic diseases [9]. Thus, these patients are less likely to be treated with appropriate antithrombotic agents. To help reduce the risk for myocardial infarctions, being able to identify the wide variety of symptoms of AF in addition to screening for AF can help physicians recognize AF early on in the disease course and initiate preventative treatment [8].

## Conclusions

The onset of AF in the setting of acute myocardial infarction represents a warning event requiring immediate intervention. Our case report highlights the significance of the bidirectional relationship between AF and myocardial ischemia. These two pathologies have common risk factors and can predispose to the worst outcome when met by coincidence. Prompt diagnosis and treatment of AF in primary care settings can prevent fatal myocardial infarction. Our case report further justifies the importance of screening for AF in primary care settings, which can tremendously reduce the burden of cardiovascular events.
